# The Impact of Asking Medical Students What They Want to Learn on Student Participation in Lectures

**DOI:** 10.7759/cureus.50970

**Published:** 2023-12-22

**Authors:** Ankur Segon, Yogita Sharma-Segon

**Affiliations:** 1 Medicine, University of Texas Health Science Center at San Antonio, San Antonio, USA

**Keywords:** curriculum development, medical education, learner engagement, attendance, acting internship

## Abstract

Background: The General Internal Medicine Acting Internship (GIM AI) at our school is a compulsory, one-month-long experience. Morning report-style case-based discussions were conducted on a weekly basis as part of the acting internship and were poorly attended. We sought to redesign our academic half day didactic curriculum and increase voluntary student attendance by allowing students to actively participate in determining the content of the acting internship academic half day.

Intervention: Prior to the beginning of the acting internship, students were sent an email survey listing seven inpatient topics to rank on a scale of 1-5 (1=not at all interested, 5=very interested). Based on student feedback, one additional topic was added: antibiotic use for common inpatient diagnoses. Topics that received the highest score were selected for topic-based sessions. A total of 32 teaching sessions were conducted over eight months. Twenty-four of these sessions were topic-based and the remainder were case-based. Student attendance at these sessions was voluntary.

Key results: Case-based discussions had the lowest preference ranking (n=94, mean=2.9), while cross-cover-based discussions (n=94, mean=4.3, p=0.001) and antibiotic use (n=52, mean=4.3, p=0.001) received the highest scores. Thirty-four percent (41/120) of possible learners attended case-based discussions, while 78% (281/360) of possible learners attended topic-based sessions (p<0.001). Learners reported a statistically significant improvement in comfort level in recognizing and managing 73% of sub-topics (29 out of 41) covered in topic-based sessions.

Conclusions: A learner-centered approach to curriculum design led to robust student engagement in our acting internship academic half day. Fourth-year students prefer specific topic-based teaching sessions over case-based, morning report-style sessions.

## Introduction

Medical students in the United States interested in an internal medicine career frequently pursue a fourth-year rotation in internal medicine. This rotation is called the medicine acting internship or the medicine sub-internship. The Alliance for Clinical Education (ACE) in its 2014 position paper recommended that students interested in internal medicine should pursue an acting internship in internal medicine [1]. The alliance also recommends that the acting internship be a required experience. The education committee of the American College of Physicians recommends all fourth-year students go through one high-intensity clinical experience during their fourth year [2]. Over 90% of medical schools offer an acting internship to fourth-year students [3]. Acting internships can be based on the general internal medicine floor, intensive care unit, or subspecialties of medicine. 

A national survey of acting internship directors in 2000 found that only one-third of acting internship courses nationwide have explicit goals and objectives [4]. To address this need, the Alliance for Academic Internal Medicine (AAIM) put together a curriculum for the internal medicine acting internship in 2002 (revised 2019) based on a needs assessment of interns, acting internship directors, and residency program directors [5]. This curriculum provides a framework to prepare fourth-year medical students for the intern year. Several topics are covered, ranging from time management and communication skills, patient care skills, and medical student wellness. AAIM also provides supplementary case studies to accompany this structured curriculum. In April 2005, Clerkship Directors in Internal Medicine (CDIM) conducted a survey of its membership in the United States and Canada to determine the current state of acting internship curricula. Thirty-five of the surveyed programs reported using the CDIM acting internship guidelines as part of their curriculum. More than 85% of respondents who used the guidelines found them to be helpful in organizing the acting internship and enhancing the quality of education. The Association of Program Directors in Internal Medicine (APDIM) and Clerkship Directors in Internal Medicine (CDIM) released a position paper in 2015 on minimum expectations of what an acting internship should look like. The position paper does not make any specific recommendations on including morning report-style case-based sessions as part of the acting internship experience but does list topics that should be covered in an acting internship. However, it remains unclear as to which topics, if any, from the CDIM curriculum should be covered in a didactic setting during an acting internship. Second, while student attendance at live lectures has been declining [6], there is evidence that student participation in lectures improves performance in school [7-10]. While incorporating active learning into lectures has been proposed as a method to improve attendance, effective strategies to improve attendance remain elusive [2].

The objectives of our study were to: (i) Elicit fourth-year medical student preferences on topics that should be covered during structured didactics on a general internal medicine acting internship, (ii) Determine if attendance at didactic sessions during the acting internship can be improved by allowing students to vote on topics covered during such sessions and by allowing students to choose which sessions to attend, and (iii) Evaluate change in students’ confidence levels in recognizing and managing topics covered during didactic sessions.

This article was previously posted to the Research Square preprint server on August 7, 2023 [11].

## Materials and methods

Thoeretical framework

The primary focus of the fourth year of medical school is to provide experiential learning through increased patient care responsibilities [12]. One of the challenges in developing structured non-clinical learning experiences for the acting internship has to do with determining the importance of such activities on a primarily experiential rotation. It is unclear what topics, if any, are regarded by fourth-year medical students as being relevant and meaningful for inclusion in an acting internship didactic curriculum. We considered Knowles’ principles of andragogy [13] and the theory of reasoned action to address this challenge. According to Knowles, adult learners are self-directed and prefer the immediacy of application rather than the future application of knowledge. This results in the development of a self-concept that is manifested by a shift from allowing others to determine what should be taught to self-reliance and independent thinking. The theory of reasoned action postulates that behavior is likely if the learner thinks that engaging in the behavior is likely to produce a desirable and valuable result [14]. Therefore, topics included in the acting internship academic half day should be immediately applicable and student-driven.

Study setting

Medicine Acting Internship (AI) is a month-long mandatory rotation at the Medical College of Wisconsin in Milwaukee, United States. Students can choose to complete their AI experience in general internal medicine, pediatrics, or family medicine wards. Fifteen students rotate on the general internal medicine AI at four different sites each month. Our study was conducted over eight months resulting in a study cohort of 120 students. Students from all four sites travel to the main site for didactic sessions. Didactic sessions, hereby referred to as “academic half day”, are offered weekly. Historically, such academic half days were 60-75 minutes in duration and covered morning report-style case-based topics where one student presented a case, and a facilitator would walk the group through the differential diagnosis and management of the case. The attendance at these voluntary sessions was anecdotally reported at 40-50%. We redesigned our AI academic half day at the beginning of the academic year by soliciting student input into the selection of topics. 

Survey development

Two weeks prior to the beginning of the AI, we emailed enrolled students a survey asking them to rate “topic-based sessions” and “case-based sessions” on a scale of 1 to 5 (1=not at all interested, 2=somewhat not interested, 3=neutral, 4=somewhat interested, 5=very interested). Case-based sessions were described as morning report-style sessions where students would bring their own cases for discussion. Six topic-based sessions were initially included in the survey: arterial blood gas (ABG) interpretation, electrocardiogram (EKG) interpretation, approach to common cross-cover scenarios, approach to common inpatient medicine diagnoses, radiology overview, and discussion of inpatient medicine specific multiple-choice questions pertinent to United States Medical Licensure Examination step 2-clinical knowledge exam. We also surveyed participants on their preference for morning report-style case-based sessions. Brief descriptors were provided as part of the survey so students would have a sense of what each session would entail. These topics were chosen based on the CDIM curriculum and input from local leaders in undergraduate medical education at our institution. The survey allowed students to write topics that were not listed on the survey. Based on consistent student feedback during the first three months, we added an eighth session to the survey: approach to using antibiotics. 

Implementation and evaluation

Each month, we chose three top-voted topic-based sessions for inclusion in the academic half day. Most of the topics were taught by the course director for the first three months. By the end of the year, four different faculty members, including the course director, were teaching these topics. Interactive, case-based, small-group sessions were designed to teach each topic-based session. Each topic-based session was taught for one to three hours. The four academic half days each month were randomly assigned to topic-based sessions or case-based sessions in a 3 to 1 ratio. Therefore, three topic-based sessions and one morning report-style case-based session were conducted each month. The topic that received the highest rating by students was taught on the first of three days assigned to topic-based sessions, and topics with the next two highest ratings were taught on the second and third topic-based session days. All students were asked to come prepared with a case to present for the case-based session. A faculty member served as the moderator and led the discussion. We conducted a total of 32 academic half days on the AI over eight months. A total of 120 students rotated through the acting internship over these eight months. We did not conduct academic half day sessions for four months in the year due to low student enrollment in the course during December, January, May, and June. 

We framed attendance at academic half day as recommended but not required. The course director explained to the students how academic half day content was determined during the monthly course orientation. The course director recommended to the students that they attend all sessions. However, students were given agency over attending by giving them the choice to skip an academic half day if they had a learning opportunity on the wards that they felt was more conducive to their learning. Students were asked to send an email to the course coordinator stating their reason for not attending in one to two sentences. Students were excused from attending on their day off and post-night call day. Attendance at each academic half day was tracked by asking students to sign in upon arrival. 

For topic-based sessions, students rated their comfort level in recognizing or managing the various sub-topics covered in the session both before and after the session on a five-point scale (1=not at all comfortable, 2=somewhat uncomfortable, 3=neutral, 4=somewhat comfortable and 5=very comfortable). Sub-topics covered during each session were determined by the acting internship director and were informed by the CDIM curriculum [1]. Paper forms were handed to students at the beginning of the session and collected at the end.

The mean score on the five-point Likert-type response scale was used to illustrate student preference for topics to be covered during the academic half day. The mean rating for case-based sessions was compared to each topic-based session. At the end of each session, students were asked to complete a session evaluation form. Before and after each topic-based session, students were asked to rate their comfort level in recognizing and managing each sub-topic covered during the session. Data was analyzed using a student’s t-test when comparing attendance at case-based and topic-based sessions and when comparing student evaluation of case-based and topic-based sessions. Paired t-tests were used when measuring a change in student comfort level with recognizing and managing the various topics covered during the academic half day. A p-value of less than 0.05 was considered statistically significant. 

## Results

Ninety-three students responded to the survey on determining academic half day content over eight months (response rate 77.5%). Traditional case-based, morning report-style sessions received the lowest rating of all eight options offered to students (mean score 2.9/5). All topic-based sessions scored higher than case-based sessions in the survey (Table 1). There was no statistically significant difference in scores between the various topic-based sessions, with a range of 3.8/5 to 4.3/5. The approach to antibiotic usage and approach to cross-cover scenarios received the highest score (4.3/5) of all topic-based sessions (Table 1). 

**Table 1:** Student scores, academic half day topics *p-value compares “case-based sessions” to “topic-based sessions”; 1-5, 1=strongly disagree, 5=strongly agree

Academic half day session	Mean score on student survey (n=93) *
Case-based sessions	2.9/5
Approach to using antibiotics	4.3/5 (p <0.001)
Approach to cross-cover scenarios	4.3/5 (p<0.001)
Approach to EKG interpretation	3.9/5 (p<0.05)
Approach to common inpatient diagnoses	3.9/5 (p<0.05)
Radiology overview	3.9/5 (p<0.05)
Approach to Arterial Blood Gas (ABG) interpretation	3.8/5 (p<0.05)
Step 2 CK based questions	3.8/5 (p<0.05)

A total of 24 topic-based and eight case-based academic half days were conducted. Thirty-four percent (41/120) of possible learners attended case-based sessions, while 78% (281/360) attended topic-based sessions (p <0.0001). Learners reported a statistically significant improvement in comfort level with recognizing or managing 71% of the sub-topics discussed (29 out of 41 sub-topics) (Table 2) during topic-based sessions. Student evaluations of topic-based sessions were statistically significantly higher than case-based sessions (Table 3).

**Table 2:** Student comfort level, before and after attending academic half day

Session	Pre-session Comfort Level	Post-session Comfort Level	p-value
Session 1: Approach to using antibiotics			
Topic	Comfort level with using antibiotics, pre-session	Comfort level with using antibiotics, post-session	
Penicillins	3.1	4.1	0.008
Carbapenems	1.9	2.4	0.12
Cephalosporins	2.9	3.8	0.01
Fluoroquinolones	2.8	3.2	0.4
Macrolides	3.1	3.9	0.07
Tetracyclines	2.4	3.2	0.27
Vancomycin	2.6	3.9	<0.001
Session 2: Approach to cross-cover cases, part 1			
Topic	Comfort level with managing, pre-session	Comfort level with managing, post-session	
Hypokalemia	3.4	4.3	0.02
Hyperkalemia	2.5	3.7	0.001
Hypocalcemia	3.1	4.2	0.005
Hypomagnesemia	3.0	4.1	0.005
Hypophosphatemia	2.8	3.6	0.04
Session 2: Approach to cross-cover cases, part 2			
Respiratory depression	2.4	3.0	0.07
Hypoxia	2.9	3.8	0.005
Hypotension	3.1	4.0	0.005
Hypertension	3.3	4.3	<0.001
Bradycardia	2.9	4.0	<0.001
Session 3: Approach to EKG interpretation			
Topic	Comfort with interpretation, pre-session	Comfort with interpretation, post-session	
Sinus bradycardia	3.8	4.2	0.3
Sinus tachycardia	3.5	4.3	0.04
Sinus arrhythmia	3.2	4.4	0.03
Multifocal atrial tachycardia	1.9	2.4	0.1
Atrial fibrillation	2.6	3.9	0.01
Premature atrial complex	2.8	4.0	0.02
Premature ventricular complex	3.0	3.9	0.035
Non-sustained ventricular tachycardia	2.1	2.7	0.09
Paroxysmal atrial tachycardia	1.7	3.1	0.01
Atrioventricular nodal reentrant tachycardia	1.8	3.2	0.02
Third-degree heart block	2.2	3.6	0.02
Session 4: Approach to common inpatient medicine diagnoses			
Topic	Comfort with management, pre-session	Comfort with management, post-session	
Cellulitis	3.2	4.1	0.008
Urinary tract infection	3.3	4.5	0.001
Community-acquired pneumonia	3.0	4.3	<0.001
Nosocomial pneumonia	2.8	3.8	0.004
Acute exacerbation of COPD	2.9	3.6	0.02
Sepsis	2.4	2.9	0.14
Asthma exacerbation	3.1	4.0	0.008
Upper GI bleeding	3.1	3.5	0.18
Lower GI bleeding	2.6	3.4	0.005
Session 5: Approach to ABG interpretation			
Topic	Comfort with interpretation, pre-session	Comfort with interpretation, post-session	
Metabolic acidosis	2.0	3.1	0.005
Metabolic alkalosis	1.9	2.4	0.3
Respiratory acidosis	2.4	3.7	<0.001
Respiratory alkalosis	1.7	2.1	0.3

**Table 3:** Cumulative student evaluation of all sessions *1-5, 1=strongly disagree, 5=strongly agree

Statement	Topic based session* (N=234)	Case based session* (N=41)	p-value
This session was helpful to my learning	4.1	3.2	<0.001
This session was applicable to me	4.0	3.1	<0.001
This session should continue to be offered	4.3	2.9	<0.001

## Discussion

This study found a much higher rate of student participation in the topic-based sessions that students voted to include in the internal medicine acting internship academic half day compared to case-based sessions. Our findings suggest that involving learners in planning session content can help increase attendance. While learner participation in curriculum design is well supported by educational theory and recommended as best practice to promote student engagement and retention [15-17], there is limited data on the impact of student engagement in curriculum design [18]. We only found one study from a law school that found improved student attendance and pass rates when students were involved in the re-design of a course module [19]. Our study provides a real-life example of improving student participation by tailoring session content to student votes.

The list of topics generated by this study can be helpful to programs when creating a dedicated academic half day or other lecture series for fourth-year medical students. We found that sessions on covering cross-cover scenarios and antibiotic selection for common diagnoses were highly rated and consequently taught every month. Of the remaining five topics, the approach to common inpatient diagnoses was highly rated in the first half of the year (July-December), and the approach to ABG and EKG interpretation, step 2 CK questions, and radiology interpretation were highly rated in the second half of the year (January-June). This was likely due to more students interested in internal medicine rotating on the acting internship in the first half of the year. 

It was challenging for the medicine acting internship director to conduct all academic half day sessions on their own while juggling clinical duties. Our series ran smoothly once other faculty were recruited to conduct six of eight sessions on a rotating basis. The AI director created PowerPoint slides (Microsoft Corporation, Redmond, Washington, United States) and handouts for all eight sessions and these were shared with other faculty.

Case-based sessions were consistently ranked lower than topic-based sessions by our students, and attendance was lower at case-based sessions. This could be due to the specialty-specific nature of case-based sessions. Since the medicine acting internship is a required rotation at our institution, many students on the rotation are interested in a career in specialties other than internal medicine. Topic-based sessions covered topics broadly applicable to many different specialties and were more appealing to our students. In addition, it is possible that acting internship students viewed the opportunity to learn case-based diagnosis and management on the wards more favorably than doing so in the classroom. 

We allowed students to excuse themselves from the academic half day if they felt they had access to better learning opportunities in the wards, if it was an off day for them, or if they were on night call the previous night. Twenty-two percent (79/360) of possible student attendees did not attend topic-based sessions. Of these, 54% (43/79) were either off or post night call on the day of the academic half day. The remainder of the students sent an email to indicate they preferred to stay in the wards and learn. Therefore, 90% of possible students voluntarily attended topic-based academic half day sessions. Our results support previous findings that mandatory attendance policies may reduce students’ motivation to attend teaching sessions by reducing their perception of control over their environment [20]. 

We tried to identify reasons behind the statistically non-significant improvement in comfort level with 29% of sub-topics taught during topic-based sessions. While this could reflect a true lack of improvement in comfort level or a lack of statistical power, we found that almost all of these sub-topics were discussed in the second half of the session. Student fatigue might have contributed to the limited improvement in comfort levels reported by students with these sub-topics. 

This is a single-center study conducted at a large, free-standing, private medical school in the Midwest United States with a class size of around 200 students. Our approach of asking students to vote on topics they want to learn about comes with challenges. The biggest challenge in the implementation of such a program lies in keeping up with the month-to-month variability in content preference as determined by student voting. Scheduling and conducting sessions became easier once we had a set of topics that started to get identified repeatedly on student voting and a pool of facilitators helping to conduct sessions. Recruiting a cohort of interested faculty upfront to design and teach these sessions can help mitigate this challenge. We did not collect data on student characteristics including their residency specialization preferences. Doing so in a future study can help identify topic preferences for students interested in internal medicine versus students interested in other specialties and allow for further customization of session content. Finally, student attendance at teaching sessions can be biased by the reputation of the attending physician conducting the session instead of the topic being covered. This is less likely in our study as the same core group of attending physicians conducted both types of sessions.

## Conclusions

Giving students a say in what will be taught during a didactic session can help improve student attendance. Fourth-year medical students in Medicine AI prefer to learn about topics that are readily applicable to patient care instead of discussing individual case presentations. Sessions delivered as part of our academic half day can be a good starting point for other institutions looking to design academic half days for their Medicine AI.
